# Community-Generated Recommendations Regarding the Urban Nutrition and Tobacco Environments: A Photo-Elicitation Study in Philadelphia

**DOI:** 10.5888/pcd10.120204

**Published:** 2013-06-13

**Authors:** Elizabeth A. FitzGerald, Rosemary Frasso, Lorraine T. Dean, Terry E. Johnson, Sara Solomon, Eva Bugos, Giridhar Mallya, Carolyn C. Cannuscio

**Affiliations:** Author Affiliations: Elizabeth A. FitzGerald, Rosemary Frasso, Eva K. Bugos, University of Pennsylvania, Philadelphia, Pennsylvania; Lorraine T. Dean, Terry E. Johnson, Sara Solomon, Giridhar Mallya, Philadelphia Department of Public Health, Philadelphia, Pennsylvania.

## Abstract

**Introduction:**

Overweight, obesity, and tobacco use are major preventable causes of disability, disease, and death. In 2010, 25% of Philadelphia adults smoked, and 66% were overweight or obese. To address these health threats, the Philadelphia Department of Public Health launched Get Healthy Philly, an initiative to improve the city’s nutrition, physical activity, and tobacco environments. The objective of this assessment was to identify residents’ perspectives on threats to health and opportunities for change in the local food and tobacco environments.

**Methods:**

Participants (N = 48) took photographs to document their concerns regarding Philadelphia’s food and tobacco environments and participated in photo-elicitation interviews. We coded photographs and interview transcripts and identified key themes.

**Results:**

Participants proposed interventions for nutrition 4 times more often than for tobacco. Participants spontaneously articulated the need for multilevel change consistent with the ecological model of health behavior, including changes to policies (food assistance program provisions to encourage healthful purchases), local and school environments (more healthful corner store inventories and school meals), and individual knowledge and behavior (healthier food purchases). Participants often required interviewer prompting to discuss tobacco, and they suggested interventions including changes in advertising (a local environmental concern) and cigarette taxes (a policy concern).

**Conclusion:**

Participants were well versed in the relevance to health of nutrition and physical activity and the need for multilevel interventions. Their responses suggested community readiness for change. In contrast, participants’ more limited comments regarding tobacco suggested that prevention and control of tobacco use were perceived as less salient public health concerns.

## Introduction

Tobacco use and conditions related to overweight and obesity are the 2 leading causes of preventable death in the United States (1,2). Of the 10 largest United States cities, Philadelphia in 2010 had the highest prevalence of smoking among adults (25%) and among the highest prevalences of regular smoking among youth (3,4). Most (66%) adults in Philadelphia in 2010 were overweight or obese, as were 41% of youth (3). To address these health threats, the Philadelphia Department of Public Health (PDPH), with funding from the Centers for Disease Control and Prevention, launched Get Healthy Philly, an initiative to improve the city’s nutrition, physical activity, and tobacco environments (5–7).

This article draws on the concept of lay epidemiology — how members of communities frame health threats in the context of their neighborhoods and their lives (8). Lay epidemiologic perspectives can inform interventions that are responsive to local concerns, are likely to be accepted and adopted by the target population, and ultimately are likely to improve health (9,10). Successful interventions consider community preferences, priorities, and readiness for change (11–14).

Most Americans think obesity and smoking are major health concerns (15), but does this perception translate into public support for preventive interventions? We report results of a photo-elicitation project that sought Philadelphians’ perspectives on threats to health and opportunities for change in the local food and tobacco environments (16,17). Photo-elicitation is a research technique through which participants are asked to photograph aspects of their lives and environments and are then interviewed by the researcher about their photographs. The overarching objective was to identify promising interventions that align with residents’ priorities and concerns.

## Methods

### Sample and procedures

Via e-mail or telephone, we recruited a convenience sample of Philadelphia residents from July 2011 to February 2012. We contacted 26 people from an outreach list provided by PDPH; 16 of those original 26 people participated, and 10 were lost to follow-up. The outreach list included organizations that focused on various health and social issues and target populations across Philadelphia.

Beginning with these 16 primary recruits, we used purposive and snowball sampling methods (18) to engage 12 additional adults and 20 youth participants aged 15 to 17 years, for a total of 48 participants (28 adults and 20 youths). Snowball sampling was originally used to facilitate rapid recruitment and provide timely feedback to PDPH as Get Healthy Philly interventions were under way. We then reviewed early participants’ characteristics and sought to maximize diversity by race/ethnicity, sex, neighborhood of residence, and age. All participants read and spoke English and were mentally competent to provide consent. For youth participants, parental consent and youth assent were required. Adult participants received no compensation for participation; each youth participant received $10 upon completion of training and $20 upon completion of the interview. Institutional review boards of the University of Pennsylvania and the PDPH approved this project.

The training session incorporated informed consent of participants, instruction on project goals and photo-elicitation techniques, and the ethics of community-based photography (19). Participants were given cameras and instructed to document their daily lives in still photographs over the following week, focusing on their nutrition and tobacco environments and related health concerns. Participants were asked to select approximately 10 photographs from each of their collections to discuss during the photo-elicitation interview (20). In the training, nutrition and tobacco use were presented as equally important domains for participant input.

### Data collection and analysis

Each of the 48 participants was interviewed in a semi-structured, photo-elicitation format based on 4 key points regarding each photograph (21):

1. Tell me the story of this photograph.
2. How does this relate to your health and the health of your community?
3. Why does this problem or asset exist?
4. What interventions would help address this concern or increase the availability of this asset?

Interviews typically lasted 40 to 60 minutes and were audio-recorded and transcribed verbatim. QSR NVivo 9 (QSR International Pty Ltd, Doncaster, Victoria, Australia) was used for data coding, management, and analysis. Data analysis began early in the project and continued in an iterative fashion throughout the assessment (22). We used an inductive coding approach in which the code structure was developed to reflect the insights of participants themselves. After the initial 8 interviews, the evaluation team developed a codebook of 22 items, based on line-by-line reading of the content (22–24). Key themes were described in memos and discussed by the study team throughout the analysis process. We reached saturation (no new themes emerged) after 48 participants completed the study. Photographs were coded for content.

**Figure 1:**
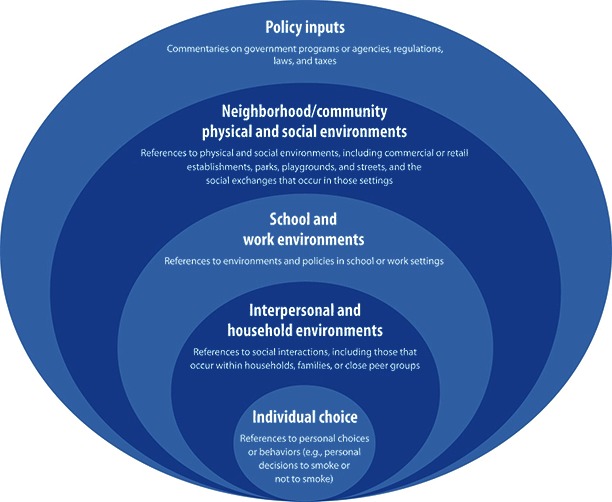
The ecological model, adapted to reflect health influences on tobacco use and nutrition in both youth and adults.

After coding all interviews and photographs, we further analyzed 2 information-rich codes: prevention strategies to improve nutrition and promote physical activity and prevention strategies to reduce tobacco use. We observed that many of the suggested prevention strategies corresponded to an ecological model of health behavior, so we further coded transcripts using this model to explore how participants discussed health risks, assets, and potential interventions. Therefore, the theoretical frame used in this article reflects both participants’ insights and elements of existing theories, including the stages of change/Transtheoretical Model (25), the ecological model, and the Health Belief Model (26). Figure 1 shows our adapted version of the ecological model.

## Results

Of 58 participants (34 adults and 24 youth) who were recruited, consented, and trained, 10 participants were lost to follow up (ie, 3 adults and 4 youth discontinued participation after training, and 3 adults did not respond to any of at least 5 attempts to schedule the interview). The final sample included 48 participants (28 adults and 20 youths) who were diverse in terms of age and race/ethnicity (Table 1) and who resided in various neighborhoods. Of the 28 adult participants, only 3 reported that they were current cigarette smokers; 15 adults and 12 youths discussed a relative or friend who smokes.

**Table 1:** Demographic Characteristics of Adults and Youths Who Participated in Photo-Documentation and Photo-Elicitation Interviews Regarding Philadelphia’s Food and Tobacco Environments (N = 48), 2012

Characteristic	Total No.
**Sex**	
Female	29
Male	19
**Race**	
White	26
African American	15
Asian	6
Other	1
**Ethnicity**	
Non-Hispanic	42
Hispanic	6
**Age, y**	
15–17	20
18–25	9
26–40	8
41–60	9
61–80	2
**Self-rated health**	
Excellent	13
Very good	26
Good	6
Fair	2
Poor	0
Not reported	1

Interview and photographic data disproportionately focused on nutrition and physical activity, with fewer photographs, less commentary, and less diverse potential prevention strategies regarding tobacco use. In their interviews, participants made 116 references to 18 different potential intervention strategies to improve nutrition and physical activity; they made only 26 references to 8 different potential interventions to reduce tobacco use. These counts include proposed interventions that were mentioned multiple times by individual participants or by more than 1 participant.

**Figure 2:**
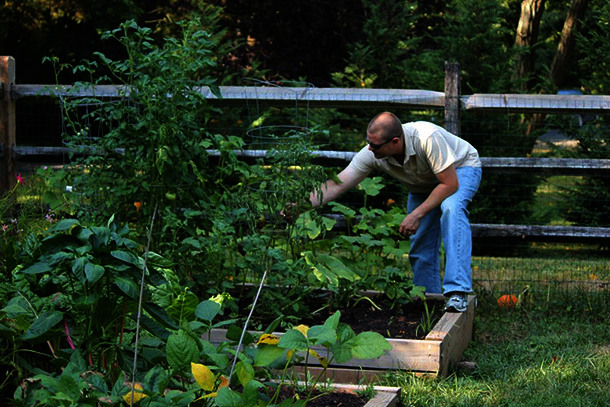
Urban gardening was described as a promising strategy to promote improved nutrition for both youth and adults.

**Figure 3:**
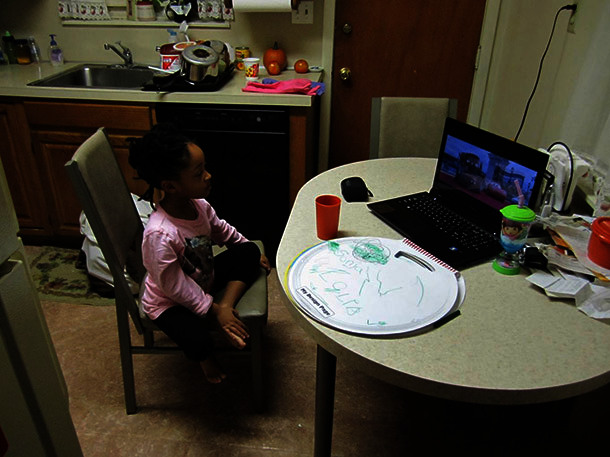
Parents described many concerns, including their frustration that it is expensive and time-consuming to prepare healthy meals and their worries that neighborhood conditions are unsafe for active outside play.

**Figure 4:**
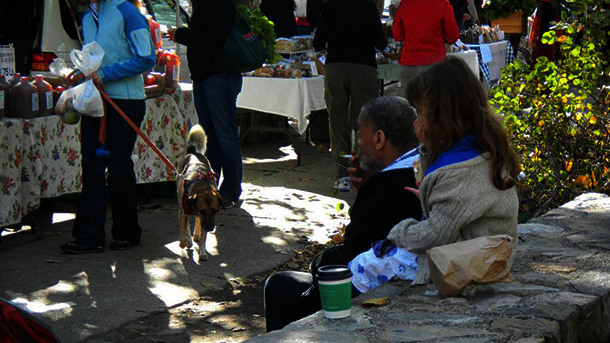
The photo-documentation strategy allowed participants to observe contradictions within the environment — including this image of ambient cigarette smoke at an otherwise health-promoting farmer’s market.

**Figure 5:**
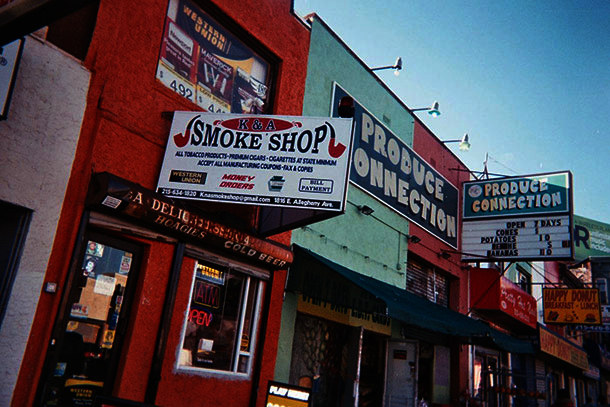
Multiple participants photographed tobacco outlets and lamented how common they are across the city.

**Figure 6:**
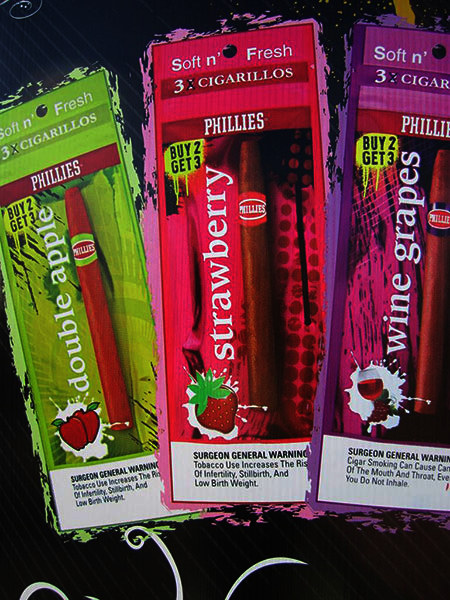
Participants described their concern — and in some cases their anger — about marketing of tobacco to youth.

**Figure 7:**
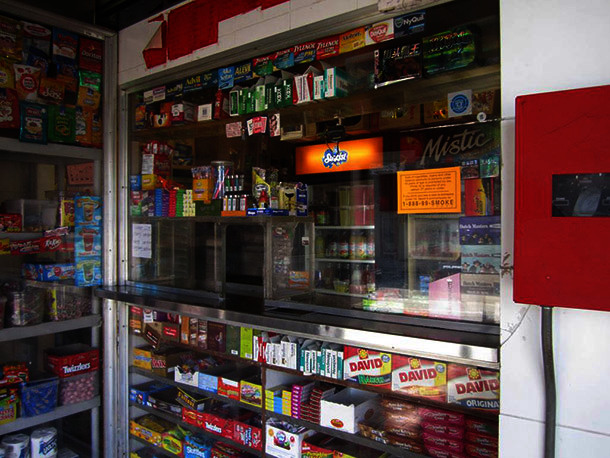
Philadelphia residents often complained about the sale of “loosies” — single cigarettes that are cheap enough for young people to purchase, offering an inexpensive introduction to tobacco.

This emphasis on nutrition was also reflected in the content of participants’ photographs. Of the 580 photographs submitted, 459 (79%) included nutrition-related and physical activity–related content (Figures 2 and 3), and only 121 (21%) related to tobacco (Figures 4–7).

Without specific prompts from the interviewer, participants spontaneously described health concerns in terms that were consistent with an ecological model. This was especially true in participants’ photographs and commentary regarding nutrition and physical activity. For nutrition and physical activity, participants proposed interventions that were equally distributed across all levels of the ecological model. Participants proposed tobacco-use–related interventions less often, and the proposals were concentrated in local community and neighborhood (23%) and policy (50%) levels of the ecological model (Tables 2 and 3).

**Table 2:** Participant-Generated Intervention Strategies to Promote Healthy Eating or Physical Activity, Classified Into Categories That Correspond to the Ecological Model, Philadelphia, 2012

Theme	Participants’ Insights on Nutrition and Physical Activity
**Individual level:** Personal preferences and constraints drive choices and behaviors; knowledge is not necessarily translated into healthy behavior. (n = 11)	• Personal responsibility and accountability are essential.
	• Time constraints interfere with healthful food preparation.
	• Changing habits and ingrained preference for unhealthful foods is difficult.
**Interpersonal level:** Interactions, including those that occur within households, families, or peer groups, contribute to the learning and adoption of healthy or unhealthy behaviors. (n = 21)	• Parent–child interactions offer both positive and negative modeling of eating habits.
	• Family mealtimes can foster communication and set tone for healthful eating.
	• Ideally, school and home environments should reinforce messages about healthful eating; when messages are conflicting, then positive efforts in 1 setting may be undermined by negative forces in the other.
**School and work level:** Environments and policies at schools and workplaces have the potential to markedly influence health behaviors. (n = 29)	• Students and adults spend a substantial proportion of their time at school and work — so it is important to maximize healthy exposures and minimize unhealthy exposures there.
	• School cafeteria food is widely critiqued by students as both unhealthful and unpalatable.
	• Students and adults are intrigued by curricular innovations around food production and preparation (eg, urban gardening).
	• Money spent on junk food takes away money for healthful food.
**Neighborhood and community level:** Neighborhoods and communities need better access to healthful foods and amenities and less access to unhealthful ones; residents need to be encouraged to use existing programs and resources. (n = 35)	• Communities have to organize to demand access to more healthful food and less unhealthful food.
	• Simple solutions, like reviving play, can be good solutions.
	• “Food culture” has to change. Often healthful food options are not “sexy” and need to be made more appealing.
	• People will not go out of their way to be healthy; make it easy for them to make healthful choices.
	• Communities have a range of underused health-promoting resources now — like parks and exercise programs.
**Policy input level:** Government programs or agencies, regulations, laws, and taxes all can be used more effectively to promote health. (n = 20)	• Urban residents are often unaware of existing programs from which they could benefit, like Philly Bucks, which provides Supplemental Nutrition Assistance Program (SNAP) recipients a $2 voucher for every $5 spent at farmers’ markets.
	• Programs that introduce children to fresh, local foods can shape healthier preferences.
**Multilevel:** Interventions should target several levels concurrently. The participant insight at right combines interpersonal and school levels; other participants combined individual, community, and policy levels.	**• “**Half of their day is in school, half of their day is at home . . . and even if they get it from the schools, but it’s not enforced at home . . . all the work that the schools do will be thrown out the window. Or if the families are the really strong one, and they eat healthy, and they get to school and then they’re given chicken fingers . . . then it messes up the balance of wanting to live a healthier lifestyle. . . . At the end of the day it’s everybody that plays a huge part in whether this child is going to be healthier and be more conscious of what they’re eating.” (African American woman, 22 years old)

**Table 3:** Participant-Generated Intervention Strategies to Prevent Tobacco Use, Classified Into Categories That Correspond to the Ecological Model, Philadelphia, 2012

Theme	Participants’ Insights on Smoking and Tobacco
**Individual level:** Personal preferences, addictions, and psychological dynamics drive tobacco use. (n < 5)	• Smokers ignore messages about health hazards or find a “thrill” in defying health warnings.
	• Pleasure-seeking is a powerful motivator.
**Interpersonal level:** Peers and role models can have an influence on tobacco use. (n < 5)	• Smoking behaviors can be transmitted intergenerationally, especially “if you see people who are smoking that maybe you know or look up to.”
**School and work level:** School-based educational programs may have a role in preventing tobacco use prevention. (n < 5)	• Only 1 person, a youth, mentioned the role of antitobacco education in schools as a potentially effective strategy for prevention.
**Neighborhood and community, social environment level:** Changes in the physical and social environments, including commercial or retail establishments, parks, playgrounds, and streets, have a role in tobacco use prevention. (n < 5)	• Banning smoking in restaurants has fostered a shift, making tobacco “less part of the culture.”
	• Changes in the physical environment — like “no smoking” signage at recreation centers and elsewhere can generate stigma around smoking and therefore discourage tobacco use.
**Policy input level:** Government programs or agencies, regulations, laws, and taxes can be used to reduce tobacco use. (n = 13)	• The city could do a better job of enforcing rules and fines for illegal tobacco sales.
	• Taxes should be used to make smoking “prohibitively expensive.”

### Participant-generated nutrition and physical activity interventions

At the individual level, an often-repeated theme was the perception that individuals have adequate knowledge regarding nutrition and physical activity, but that this knowledge is not translated into healthy behavior. One participant mentioned the importance of individuals “really taking that initiative. It’s not someone else’s responsibility — it’s your health, your responsibility” (African American woman, 30 years old). Participants also mentioned the need for youth to “take control of their own lives, instead of saying, you know, ‘I don’t have this, I don’t have that’” (African American woman, 30 years old).

At the interpersonal level, both youth and adult participants considered parents to be influences on youth behavior and recommended strategies emphasizing parents’ role. Respondents indicated that eating behaviors are often passed down through generations within families, one mentioning the “generations of junk food” perpetuated within the household (white woman, 43 years old). Participants also recommended encouraging healthful grocery store shopping and helping time- and budget-constrained parents prepare healthful meals at home. One participant noted, “Parents are really busy, and I’m not faulting them, but by the same token too, you know from all the research, that if you sit down and have a meal with your family, it makes a big difference” (white woman, 50 years old).

Participants saw school and work environment interventions as particularly important because of the time spent in these settings. They suggested using school and work to expose people to new, healthful foods that they might not try elsewhere and “eliminat[ing] all the junk food” so that people would “be forced to eat healthy” (white male youth, 16 years old). Sixteen of the 29 participants who mentioned school- or work-related interventions specifically mentioned improving school lunches as essential. One youth participant lamented that “most of the time I don’t eat lunch because sometimes it’s not even healthy for me” (Hispanic male youth, 17 years old).

Reflecting on neighborhood and community physical and social environments, participants emphasized encouraging residents to use community resources like urban gardens and exercise clubs. Many also thought that it was important not only to bring fresh fruits and vegetables into neighborhoods that lack fresh, healthful foods (through expanded access to farmer’s markets and healthy corner stores) but also to change the urban food culture. One participant spoke about energizing the community and “opening a dialogue,” so that “people are saying, ‘Look, we want healthier foods in our neighborhoods. What are you going to do about it?’” (African American woman, 30 years old). Other suggestions included making healthful foods more visually appealing (eg, attractive displays in corner stores), and removing sources of unhealthful foods, creating a “desert for fast food” (African American woman, 28 years old).

At the policy level, participants identified the importance of government-funded initiatives. They wanted improvements in, and better promotion of, programs like Philly Food Bucks, which allows Supplemental Nutrition Assistance Program (SNAP) participants to receive a $2 coupon for every $5 spent at participating farmers markets. Finally, several participants mentioned tax policies that would discourage unhealthful eating, like a sugar-sweetened beverage tax, and giving “corner store and bodega owners an incentive to sell healthy fresh fruits and vegetables” (African American woman, 30 years old).

### Participant-generated tobacco interventions

Although participants proposed fewer and less diverse tobacco use interventions overall, they did recommend interventions at multiple levels of the ecological model (Table 3). Interviewers noted slight reticence and hesitation from many participants when discussing tobacco in general, which may have contributed to the lower number of tobacco interventions recommended in the project.

At the individual level, participants noted that the pursuit of pleasure and the addictive nature of cigarettes motivate many smokers. Participants were troubled that smokers may feel immune to the negative health effects associated with smoking and ignore health warnings. A youth participant noted that, because there is so much information available about the negative health effects of smoking, “when people do smoke now, there’s the thrill of . . . doing something dangerous” (Asian man, 19 years old).

At the interpersonal level, participants noted that exposure to family members’ (especially parents’) smoking may encourage children to adopt the same behavior. Several adult and youth participants hoped that parents would become more active antismoking advocates. Only 1 participant, a youth, mentioned drug and alcohol resistance programs and education in schools as an effective prevention strategy.

At the neighborhood and community level, participants recommended changing the culture surrounding cigarettes and promoting “the negative stigma that’s associated with smoking” (Asian man, 19 years old). Ideas included creating smoke-free environments in neighborhoods and advertising the negative health effects of smoking in community settings, to “[scare] . . . smokers to at least try to quit” (white male youth, 17 years old). One participant recommended educating tobacco retailers to prevent the sale of tobacco to youth.

At the policy level, participants wanted to make tobacco “prohibitively expensive” to discourage people from smoking (white man, 57 years old). Participants also wished to see stricter enforcement of laws against selling to youth and selling individual cigarettes (“loosies”). Participants appreciated Philadelphia’s efforts to make recreation centers and restaurants smoke-free, and recommended expanding these efforts to ban smoking in more public spaces.

### Lay applications of the ecological model

Within the 116 references to nutrition and physical activity interventions, participants discussed a range of potential solutions, which were broadly distributed across all levels of the ecological model. This framing was not as consistent for the 26 responses relating to tobacco (Tables 2 and 3).

Not only did participants see poor nutrition as a complex issue requiring multilevel interventions but several participants explicitly stated that interventions should simultaneously address those multiple levels of influence. Without prompting from the interviewer, several participants described how the levels of the ecological model can be mutually reinforcing — or undermining. For tobacco, no participants mentioned interventions that would simultaneously target more than 1 level of the ecological model.

## Discussion

The photography-based methods used in this project provided insights into participants’ beliefs and priorities, generating a range of potential interventions for consideration by community-based organizations and health departments. The quantity, complexity, and diversity of responses relating to nutrition and physical activity may reflect the salience of these health concerns to participants, especially when contrasted with the lower frequency of tobacco-use–related discussions.

Many of the interventions recommended by participants correspond with the activities of Get Healthy Philly, an effort to promote healthful eating, active living, and a smoke-free existence. In partnerships with community-based organizations, government, academia, and the private sector, Get Healthy Philly has intervened at multiple levels of the ecological model. Efforts (7,27) include increasing access to healthful foods for low-income residents through farmers markets and corner stores, installing new bike lanes, and creating wellness councils in 171 public schools. An executive order made all city-owned recreation centers, playgrounds, and outdoor pools smoke-free. Penalties for illegal tobacco sales to minors were increased, and a law was passed requiring retailers to obtain a permit to sell tobacco. Other policy efforts focused on expansion of public and private insurance coverage for tobacco use cessation treatments. Two mass media campaigns — 1 increasing parents’ awareness about sugar-sweetened beverages and another encouraging smoking cessation — achieved more than 60 million impressions between December 2011 and March 2012.

The data presented in this article suggest that our participants were primed for change regarding nutrition and physical activity (25). They appeared less prepared for interventions targeting tobacco control — consistent with the “precontemplation” in the stages of change/Transtheoretical model (25). Past studies have assessed readiness to change with self-administered questionnaires, clinician ratings, and telephone interviews (28,29). This photo-elicitation study demonstrates another method to evaluate lay health priorities and a community’s potential readiness for change, as reflected by their photographs and commentary.

These data can inform interventions. Evaluators engaged in other community-based participatory assessments have reflected that considering community priorities was key to their success, while interventions that failed to account for lay explanatory models of health and lay epidemiologic priorities were less effective (8,12–14). The California Endowment’s 10-year initiative, Building Healthy Communities, offers an example of intensive up-front community engagement to identify local priorities (30).

We propose several possible reasons why participants in this study emphasized nutrition more often than tobacco. First, eating is universal and smoking is not. Only 3 of the 28 adult participants reported that they were smokers — lower than the citywide rate of 1 in 4. Participants may have unconsciously participated in the process of “othering” by distancing themselves from smokers and viewing tobacco use as someone else’s problem (31). Had the study included more smokers, there might have been a greater focus on tobacco. However, many participants (15 adults and 12 youth) discussed concerns regarding friends and family who smoked. Second, more than 80% of participants reported their self-rated health as excellent or very good. These residents, whose perceived health status was high, may have been more inclined toward healthier lifestyles than the average Philadelphian. Third, smoking has been increasingly stigmatized. Recent tobacco control initiatives — including the 2006 bill that banned smoking in Philadelphia public spaces — have capitalized on that stigma to garner public support for restrictions on tobacco sales, advertising, and use (32–34). This stigma may also have influenced our data collection. As noted in our Results section, interviewers observed that many participants, particularly youth, were reticent to talk about tobacco and sometimes appeared uncomfortable when doing so. Although stigma may work to discourage tobacco use, it may also interfere with candid conversations to understand lay epidemiologic perspectives (and preferred intervention strategies) regarding tobacco (34,35). A fourth explanation could be “issue fatigue” surrounding tobacco, accompanied by a degree of vogue around discussion of food and obesity. Public health researchers may be complicit in this change in attention: a Thomson Reuters (ISI) Web of Knowledge search, restricted to 2011, of the terms “food AND environment” and “tobacco AND environment” returned 2,541 and 277 citations, respectively. The lay commentary elicited in this study mirrors differential research and publication emphasis in the public health community.

There were several limitations of this study. First, participants may have disproportionately focused on issues that lend themselves to being photographed (eg, food purchases and food choices) while de-emphasizing less visible features (eg, social interactions). However, participants often used photographs to initiate discussion of abstract concepts, including the behaviors and social interactions they observed in their homes, schools and workplaces, and communities. Second, although we achieved a diverse sample, of the 5 adults who dropped out of the study, 4 were African American men. Our future work will emphasize strategies to oversample and retain African American men. Third, we recruited a convenience sample in order to expedite feedback to the health department to inform an ongoing set of interventions. This was not a representative sample of Philadelphians, and the results may not generalize to other populations. Finally, our sample was limited; these findings should be tested in larger representative samples.

In this study, participating Philadelphians appeared prepared to support healthy change in their city with regard to nutrition and physical activity. They appeared less activated regarding the city’s high rates of tobacco use. Steps must be taken to revive tobacco-use–related discussions in the community, framing the issues so that tobacco is on the public’s health agenda, as well as on the agenda of public health professionals. This project offers a way to understand a population’s framing of health issues, which can inform intervention strategies.
